# A qualitative comparative analysis of well-managed school sanitation in Bangladesh

**DOI:** 10.1186/1471-2458-14-6

**Published:** 2014-01-08

**Authors:** Christie Chatterley, Amy Javernick-Will, Karl G Linden, Kawser Alam, Laure Bottinelli, Mohini Venkatesh

**Affiliations:** 1 Department of Civil, Environmental and Architectural Engineering, University of Colorado, Boulder, USA; 2 School Health and Nutrition Department, Save the Children, Dhaka, Bangladesh; 3 Department of Education and Child Development, Save the Children, Washington, USA

**Keywords:** School sanitation, Handwashing, Qualitative comparative analysis, Hygiene, Sustainability, Bangladesh, Asia

## Abstract

**Background:**

Continued management of sanitation and hygiene services, post-intervention, is a global challenge, particularly in the school-setting. This situation threatens anticipated impacts of school sanitation and hygiene investments. To improve programming and policies, and increase the effectiveness of limited development resources, we seek to understand how and why some schools have well-managed sanitation post-intervention, while others do not.

**Methods:**

Based on in-depth qualitative data from 16 case schools in Meherpur, Bangladesh, we employ fuzzy-set qualitative comparative analysis to identify the necessary and sufficient conditions, or combinations of conditions (referred to as *pathways*), that lead to either well-managed or poorly managed school sanitation. We include posited sustainability determinants from the literature and factors that emerged from the cases themselves in the analysis.

**Results:**

We identified three distinct pathways sufficient to support well-managed services, providing multiple options for how well-managed school sanitation could be encouraged. Two of these are applicable to both government and non-government schools: (1) quality construction, financial community support and a champion; and (2) quality construction, financial government support, a maintenance plan and school management committee involvement. On-going financial support for operations and maintenance was identified as a necessary condition for continued service management, which was absent from many schools with poorly managed services. However, financial support was insufficient alone and other conditions are needed in conjunction, including quality construction and incentivizing conditions, such as school management committee involvement in sanitation specifically, a sanitation champion, and/or one teacher clearly responsible for toilet maintenance. Surprisingly, the number of students per toilet (ranging from 18–95 students) and toilet age (ranging from 8–32 months) had no significant effect on sanitation conditions.

**Conclusions:**

Findings corroborate those from a similar study in Belize, and comparison suggests the need for financial community support and the possibly tenuous reliance on local champions in the absence of adequate government support for operations and maintenance. Sub-determinants to the necessary conditions are also discussed which have implications for school sanitation in Bangladesh and may have broader relevance for other low-income countries though further research is needed.

## Background

As a component of quality education, school-based sanitation and hygiene interventions have the potential to boost student health and attendance [1,2]. Unfortunately, despite increasing efforts to improve school sanitation and hygiene services in low-income countries, management of services over time remains a persistent challenge that can negate anticipated impacts of investment [3,4]. Infrequent soap provision and poorly maintained toilets are often observed within a few months or years post-intervention [5-8]. The positive impacts linked with handwashing are unlikely without *reliable* access to soap as a critical first step to behavior change [2,9,10]. Similarly, dirty or poorly maintained toilets are unlikely to be used by students and are a potential health hazard if they are used [6,11,12]. Therefore, understanding what conditions promote continued management of quality school sanitation and hygiene services is needed to improve effective resource utilization and ensure lasting impact of investments.

Drivers of well-managed school sanitation and hygiene services (i.e. regular maintenance including toilet repair, cleaning, and provision of soap and water) have been posited in sector reports and manuals [13-15]. However, there is limited empirical evidence regarding how conditions influence service management, particularly collective effects [5,6]. As an example, Saboori et al. identified four conditions that were common among schools that maintained water and hygiene activities, but found the same conditions present in schools that had discontinued service provision, suggesting their insufficiency to promote well-managed services [8].

To support more effective policy and programming, there is a need to identify *sufficient* combinations of conditions (referred to as *pathways*) that consider the collective effects of conditions based on empirical data. Multiple solution pathways would also enable more flexible, practical, and economically viable options to respond to local needs and limitations. In response, a study of schools in Belize, used crisp-set qualitative comparative analysis (csQCA) to evaluate the collective effects of social and technological conditions on continued toilet maintenance [16]. The authors (which are also CC, KGL and AJW of this article) identified five pathways to well-maintained school sanitation, with local involvement upfront considered a necessary condition. However, the singular study site and exclusion of schools with "moderate" sanitation services (due to the binary nature of csQCA) may limit the generalizability of findings, and a similar study in a different geographical location that includes moderate cases is needed to expand upon findings.

In this study, we compare cases from a school-based sanitation and hygiene intervention in rural Bangladesh to identify sufficient pathways to effective service management over time. As an additional and timely research objective, we investigated the different pathways to well-managed sanitation services in government primary schools (GPS) versus registered non-government primary schools (RNGPS) which may have implications to support the continuity of well-managed services through the nationalization of RNGPS, which began in January 2013 [17].

### Research setting

In the small western district of Meherpur, educational outcomes and access to sanitation and hygiene in schools are some of the lowest in the country. A baseline study found a high dropout rate with 58% of students regularly attending primary school, and functioning sanitation and handwashing facilities at only 36% and 47% of schools, respectively [18]. In response, the non-governmental organization (NGO) Save the Children has been implementing the *Shishuder Jonno* ("For Children") program since 2007.

One component of the intervention includes the construction of sanitation and hygiene facilities at schools, and hygiene education for teachers, parents and children. Save the Children and government partners also provide health-related training and guidance to the SMC—a group of 12 community members and teachers that meet monthly to manage school activities according to government mandate. In 2012, student health clubs were also formed and trained under the title of "Little Doctors" with responsibilities including sharing hygiene messages from training sessions with Save the Children and cleaning the school toilets. Another central component of the program is the continuous support provided to schools through Save the Children field officers. Each field officer is responsible for five to seven schools that they visit multiple times per week to monitor and support the weekly health class and sanitation, hygiene and health services. The program has invested substantial resources to improve school sanitation, but on-going service management is a challenge, as expressed by one teacher who says, *"When Save the Children gave us the toilet, it was very easy to receive but to sustain it is so tough… it is harder to protect freedom than to achieve freedom."*

## Methods

We use the fuzzy-set variant of qualitative comparative analysis (fsQCA) to evaluate the conditions that are present in schools with sanitation services ranging from well- to poorly-managed. Due to the nascent usage of QCA in sanitation and hygiene research, we first provide a brief background of the method, followed by a description of the outcome of interest, case school selection, data collection, and calibration of outcome and conditions coding.

### Fuzzy-set qualitative comparative analysis

QCA is a case-comparative analytical method that combines the in-depth knowledge of case studies with the inferential power of "large-N" studies. It allows for the generalization of findings from a relatively small number of cases and offers the ability to identify different pathways of condition combinations that lead to a similar outcome [19,20]. Contrary to statistical methods, which measure the average effect of independent variables on a dependent variable, QCA compares empirical evidence with all theoretically possible combinations that could produce an outcome and considers the collective effects of those conditions. It is an iterative process that involves defining an outcome of interest, identifying conditions thought to influence that outcome through literature review and the cases themselves, quantifying and tabulating the outcome and conditions for multiple cases, and identifying patterns in the resulting table to isolate pathways of conditions that support the outcome. Jordan et al. [21] provide further details and a conceptual framework.

QCA scoring is based on set membership, where conditions and outcomes are coded based on the extent of membership in a set of cases sharing a particular characteristic. Whereas csQCA assigns binary scores of 0 and 1, fsQCA allows for ordinal or scale scoring of conditions and outcomes, permitting partial membership scores in the interval from 1 ("fully in" the set of cases with a given characteristic) to 0 ("fully out" of the set of cases with a given characteristic), with 0.5 indicating the point of maximum ambiguity where a case is neither more "in" nor "out" of the set [22]. FsQCA is well-suited for research on the drivers of effectively managed school sanitation due to (1) the likelihood that there are multiple pathways to well-managed services; (2) the challenge of operationalizing qualitative concepts such as community support within traditional quantitative measures; and (3) the difficulty in obtaining a full picture of the situation in each school for a large data set.

To aid in the analysis, we used fs/QCA 2.5 software ( http://www.compasss.org), which summarizes the information in a table of coded conditions (termed a "truth table") and uses Boolean logic, rather than correlation methods, to determine the necessity and sufficiency of conditions, and combinations of conditions, that lead to the outcome. We present the intermediate solution where solutions are simplified based on theory and case knowledge [23,24].

In QCA nomenclature, necessity and sufficiency are calculated through *consistency* measures, which evaluate the frequency with which conditions are present when the desired outcome is achieved. In the necessity calculation, conditions with a consistency score of 0.9 or higher are considered "necessary" or very common, while in the sufficiency calculation, conditions with a consistency score of at least 0.8 are considered sufficient [25]. A second measure of "goodness-of-fit" used in QCA is *coverage,* which indicates how well the necessary and sufficient conditions are represented by the empirical cases [26].

### Defining the outcome of interest

We define an outcome of well-managed sanitation services as reliably functioning (including secure doors and locks to provide privacy) and clean toilets, with water and soap available inside. These criteria are based on sector literature, where well-maintained, clean and private toilets have been associated with higher student toilet use [5,6,12,27]. We also included the presence of soap and water in the outcome definition based on recent findings from Kenya which found that the addition of new latrines to intervention schools significantly increased health risk among girls, likely due to unreliable provision of soap and water, and anal cleansing materials [3]. The presence of water inside the toilet is of particular importance in Bangladesh where water is culturally the primary anal cleansing material.

### Case selection and data sources

Schools were selected purposively based on Save the Children monitoring data, rather than randomly, to ensure variation of the outcome between cases, as suggested in QCA literature [28,29]. All schools were located in Meherpur Sadar sub-district, all within about a one hour drive from each other in a similar geographical setting, with student populations between 72 and 287 per shift. Sixteen case schools were included in the fsQCA based on the following criteria: (1) participated in the *Shishuder Jonno* program, (2) someone that was present during toilet construction is still at the school who can answer questions about the construction process, and (3) the program toilet has needed repair since construction. Any schools that had not faced repair needs for the toilet were removed from the analysis. Although schools that have never had to repair the toilet may be "sustainable", we removed these schools based on our research goal of evaluating a school’s ability to recover from a breakdown, which serves as an indicator of long-term *resilience* since breakdown at some point is likely.

With permission from the local government, we visited the schools unannounced over five weeks in June and July 2012. Qualitative information was gathered for each school through (1) semi-structured interviews with teachers and the field officer assigned to the school (separately), (2) focus group discussions with four boys, another with four girls, and a third with four "Little Doctors" from grade four or five (age 9–11), and (3) systematic inspection and photos of the student toilets. Data collection protocol was piloted at two schools previous to data collection. To select focus group participants, after presenting the general purpose of the study, students were asked to volunteer and participants were randomly selected from the volunteers. Interviews and focus groups incorporated specific questions related to sustainability factors postulated in prescriptive sector literature (Table 1). Additionally, open-ended questions allowed conditions, or specific aspects of postulated conditions, to emerge from the data collection process. All interviews and focus groups were conducted in Bangla, and recorded and transcribed to English by the interviewer. Data were then coded according to hypothesized conditions using Microsoft Excel and Word so that all authors participating in data analysis, including inter-rater reliability tests, were able to access the information through a familiar program.

**Table 1:** Conditions considered for inclusion in the analysis

**Postulated influential conditions/themes**	**Constant**	**Excluded**	**Included**
Policy environment	X		
Appropriateness of technology	X		
Vandalism	X		
External monitoring	X		
Participation in planning and construction	X		
Maintenance procedures/planning			X
Access to parts and services	X		
Access to a reliable water source	X		
Community support			X
SMC activeness and involvement in sanitation			X
Government involvement and support			X
On-going NGO support	X		
Presence of a local sanitation champion			X
Student engagement	X		
Hygiene education/promotion	X		
Students per toilet ratios		X	

Free and informed consent of the participants was obtained through a signed waiver by head teachers and verbal consent of students and other participants. The study protocol, including consent waivers and transcripts, was approved by the Institutional Review Board of the University of Colorado, USA, protocol # 0110.37 (approved 10 June 2010). The data collection and reporting adheres to RATS guidelines on qualitative research ( http://www.biomedcentral.com/authors/rats).

### Identification of conditions

Based on iterative analysis of potentially influential conditions from the literature [5,6,8,13-16], we included six conditions in the QCA (Table 1). Conditions with less than 30% variation among the case schools were considered *domain conditions* (i.e. constant conditions), and, as recommended in QCA literature, were not included in the analysis [26]. The number of students per toilet was also excluded because this was not associated with cleaner, better maintained or more frequently used toilets in the literature [5,6] or in the empirical cases. After removing conditions found in prescriptive literature that were constant between cases and allowing for emergent themes during data collection, we analyzed six conditions including: (1) high quality construction, (2) community support for maintenance, (3) government support for maintenance, (4) an active school management committee (SMC), (5) the presence of a maintenance plan for sanitation, and (6) the presence of a sanitation champion.

### Calibration of outcome and conditions

Following guidelines in QCA literature, we developed a rubric (Table 2) to assign codes for the outcome and conditions at each school based on the triangulation of interview, focus group and observational data [30]. During this iterative process, the calibration criteria were explicitly defined, emerging from the literature and the cases themselves. We operationalized the outcome of well-managed school sanitation services based on the minimum of two measures: (1) reliably functional toilets, and (2) reliably clean toilets, where a value of 1 was assigned for positive cases, a value of 0 for negative cases and 0.67 or 0.33 for cases falling in-between. Scores were based on student responses and facility inspection, with supplemental information from teachers and the assigned field officer. The minimum value of the two measures was used based on research that suggests that if toilets are not reliably functional, students are unable to regularly use them, and if they are not reliably clean, it is unlikely that students will regularly use them [5,6]. This same process was followed to operationalize each condition, as shown in Table 2.

**Table 2:** Coding rubric developed to score outcome and conditions at each case school

**Condition**	**fsQCA coding scheme**	**Source**
OUTCOME Well-managed sanitation services	Minimum of the following two measures: *Reliably functional toilets*^*a*^*:*	Students; Observation; Teachers; Field officer
	1: students have reliable access to functional services; repairs timely addressed	
	0.67: all toilets usually function, but repair needs are not always timely addressed	
	0.33: some toilets are frequently unusable; repairs are not timely addressed	
	0: students do not have reliable access; repairs are rarely addressed	
	and *Reliably clean toilets*^*b*^*:*	
	1: all toilets are almost always clean and quickly cleaned when dirty	
	0.67: usually more or less clean, with some instances where they remain dirty	
	0.33: frequently unclean and are usually considered unclean by students	
	0: rarely clean and students label them as dirty	
Quality construction	1: high quality materials and construction^c^ observed; no repair needs due to poor quality	Observation; Teachers; Field officer
	0.67: mostly high quality materials and construction observed; very minor repair needs due to poor quality	
	0.33: poor quality materials or construction observed, but so far there have been no repair needs because of this	
	0: poor quality materials or construction observed and have had major repair needs because of this	
Community support	1: community has contributed financially to toilet maintenance when needed	Teachers; Field officer
	0.67: community contributes financially, but not every time the school requests help	
	0.33: community members provide limited support, such as providing a few bars of soap	
	0: community does not contribute at all to maintenance of the toilets	
Government support	1: currently has government maintenance (SLIP) fund (app. 240–370 USD/year) and contingency fund (app. 9 USD/month)	Teachers; Field officer
	0.67: currently has SLIP fund, but not contingency fund	
	0.33: currently has contingency fund, but not SLIP fund	
	0: the school does not receive any government funding	
Active school management committee	1: Members check the school toilets or talk with students at least once per month, and manage repairs if needed	Students; Teachers; Field officer
	0.67: Members visit the school but not regularly (less than once per month) or limited in scope, but have or would manage repairs	
	0.33: Members rarely visit the school and are minimally involved in sanitation	
	0: Members don’t ever visit the school or manage repair needs	
Maintenance plan	1: a specific teacher is responsible for toilet maintenance and has a cleaning schedule which is followed/monitored	Students; Teachers; Field officer
	0.67: cleaning schedule usually followed but no specific teacher responsible	
	0: no specific teacher responsible for sanitation; no cleaning schedule or rarely followed	
Sanitation champion	1: someone voluntarily takes extraordinary interest in school sanitation & is recognized by others (without whom hygiene activities would likely diminish or discontinue)	Observation; Students; Teachers; Field officer
	0.67: someone leads sanitation activities but doesn’t include all aspects of maintenance and hygiene practices or others are identified who may continue their role	
	0.33: someone takes interest in sanitation at the school, but they don’t always take action or others would likely continue their role in their absence	
	0: There is no one identified as taking interest in sanitation at the school	

^a^"Functional" = waste is easily flushed, the building structure, doors & locks function providing privacy, water is available, and soap is available in or near the toilet; "Repairs timely addressed" = minor critical repairs (needed for use), such as a door lock or clogged toilet, are repaired within 24 hours, major critical repairs, such as a broken pan or door, are repaired within 1 week, minor non-critical repairs, such as a broken tap, are repaired within 1 week, and major non-critical repairs, such as a broken water pump, are repaired within 1 month.

^b^"Clean" = no visible feces on the floor/walls/seat, no flies, and no foul smell.

^c^Observation of construction and materials included quality of the superstructure, slab, piping, toilet pan, and door/lock. Examples of "poor quality" included: improper pan placement limiting water flow, roof caving due to poorly spaced supports, severe concrete scaling or cracking, and exposed or shallow septic tank piping.

Inter-rater reliability tests were conducted by two authors (CC and KA) independently coding the data and then discussing and comparing the calibrations to improve the clarity and reliability of the rubric and ensure that the calibrated conditions accurately reflected the cases studied [21,31]. A third author (LB) then reviewed final coding and rubric definitions. A summary of the coded data for each case is presented in Table 3. In the results, we provide further details and examples of high and low scoring cases for each condition to provide context of the range of conditions at the schools beyond the definitions listed in Table 2.

**Table 3:** **Data matrix of outcome and conditions for school sanitation management**^**a**^

**School**^**b**^	**Quality construction**	**Community support**	**ActiveSMC**^**c**^	**Government support**	**Maintenance plan**	**Champion**	**Outcome**
1 (GPS)	0	0	0.33	1	0	0	0
3 (GPS)	1	0	0.33	1	0	0	0
6 (GPS)	0	0	0.33	0.33	0.67	0	0
17 (RNGPS)	0.67	0.33	1	0.33	0	0	0
12 (GPS)	1	0.67	0	1	0	0	0.33
13 (GPS)	1	0	0.67	0.33	0.67	0.33	0.33
15 (RNGPS)	1	0	0	0	1	0.67	0.33
16 (RNGPS)	0.33	0.33	0.67	0	0.67	0.33	0.33
20 (GPS)	1	0	0.67	0.33	0	0	0.33
2 (RNGPS)	0.67	1	0	0	0	0.67	0.67
4 (RNGPS)	1	0.67	1	0.33	1	0.67	0.67
8 (RNGPS)	0.33	0	0.33	0.33	0.67	1	0.67
10 (RNGPS)	1	0.33	1	0.67	1	0.33	0.67
14 (GPS)	0.67	1	1	0.33	0.67	1	0.67
18 (RNGPS)	0.33	1	1	0.33	1	0.67	1
19 (GPS)	1	1	1	1	1	0.33	1

^a^The outcome of well (or poorly) managed school sanitation, as well as each of six potentially influential conditions, are coded for each case school based on set-theory, where 0 represents "fully out" of the set of cases with the given condition or outcome characteristics, 1 represents "fully in" the set of cases with the given condition or outcome characteristics, and 0.5 represents maximum ambiguity, meaning that a code of 0.33 indicates more out of the set than in, and 0.67 indicates more in the set than out.

^b^GPS = Government Primary School; RNGPS = Registered Non-Government Primary School.

^c^SMC = School Management Committee

## Results

### Sanitation management

Of the 16 case schools analyzed, seven were coded as having well-managed sanitation services (a score of greater than 0.5) and nine were coded as poorly managed (a score of less than 0.5). Only two schools were assigned the highest outcome score of 1. These schools have reliably functioning and clean toilets, with maintenance needs conducted in a timely manner: *"Our toilet is always kept clean. Once a month, the younger students may make the toilet dirty, but students clean it when they see it"* (focus group, boys, school 19), and *"When the soap becomes empty we ask the teacher for soap and the teacher gives it to us. One bar of soap is enough for 15 days"* (focus group, girls, school 18). On the other end of the spectrum, four schools had very poorly managed sanitation with a score of 0, where boys in the focus group discussions explained that *"When they open the toilet, the next day it becomes clogged and closes again for two weeks"* (School 6), and *"When the soap runs out the teachers don’t replace it for a month"* (School 17).

The age of the toilet facilities ranged from eight to 32 months, but there was no association between toilet age and condition (τ = -0.08, p = 0.34). The number of students per toilet was also not associated with toilet condition in the empirical cases, where ratios ranged from 18 to 95 students per toilet or urinal (τ = 0.09, p = 0.32).

### Quality construction

Schools 1 and 6 have had extensive repair needs due to poor quality construction and assigned a code of 0, as elucidated by a teacher at school 6 who says, *"We think it is because of the faulty toilet pan because all the toilets in this region which were constructed by the same contractor are having the same problem."* and the field officer for school 1 who describes the cause of clogging as *"…probably due to bad construction. This is not the only school where it has happened."* Three schools, coded as 0.33, also felt the quality was poor but did not cite this as a frequent cause of breakdown, as a teacher at school 8 explains, "*We found that the pipe was poor quality, so we think the other materials were poor quality too."* Construction quality was confirmed through observation of the toilet facilities. The majority of toilets were well-constructed with quality materials, however in the schools coded as 0 or 0.33, we observed problems such as pipes not buried deep enough in the soil, improperly spaced roof support rods, and poor plaster finishing.

### Community support

The community contributes financially to toilet maintenance when needed at four schools, coded as 1: *"When we needed to repair the motor, the local community…contributed 20% of the total cost"* (Teacher, school 14), and *"The local community helps us whenever we need. If we have a problem…they give 500, 700 or 1000 taka* (app. 6–12 USD) *among themselves"* (Teacher, school 18). At two schools, coded as 0.67, the community provides financial support, but not every time needed, such as school 4, where the head teacher says, *"Yes, they help, but minimally. For example, we have two teachers assigned by Save the Children. Besides Save the Children, we have to pay them 1000 tk* (app. 13 USD)*. In this situation, the community helps."* At the three schools coded as 0.33, the community, or someone in the community, has provided financial support, but the support is very limited or unreliable as expressed by teachers at school 10, *"The village or parents don’t contribute financially for toilet maintenance except the chairman,"* and school 16, *"…for the last two months we haven’t been able to pay the cleaner because the community stopped providing money and right now we have no [SLIP] fund."* The community does not support sanitation maintenance in any way at seven schools, coded as 0: *"The villagers don’t contribute to toilet maintenance…. Even when we ask the students to bring their exam fees (10-15tk), we have to face questions from 70% of the parents"* (Teacher, school 3).

### Active SMC

Most schools have an "active" SMC in the sense that they meet monthly and the majority of members attend the meetings: the SMC at 14 of 16 schools have met every month for the previous six months and at least seven of 12 members attended the last three meetings at 11 schools. However, there are still schools where meeting attendance and frequency are low, such as school 3 where the SMC met only three times in the previous five months with an average of four to five members at the last three meetings. Based on the case data, all SMCs that are highly involved in school sanitation meet monthly with at least eight members. However, meeting frequency and attendance do not guarantee sanitation activity; the SMC at schools 12 and 15 meet every month with nine and eight members on average, respectively, yet neither is involved in sanitation at the school. In this sense, the criteria for SMC activity emerged from the cases themselves. Specifically, the importance of SMC involvement in sanitation and hygiene emerged as a stronger indicator than meeting frequency and attendance. For this reason, we coded the SMC at each school based on their specific involvement in school sanitation, regardless of meeting frequency or attendance.

Six schools were coded as 1, where SMC activities include sanitation, such as monitoring the toilets, talking with students and/or parents about toilet use or handwashing, and managing maintenance needs: *"Now [the SMC] are building a boundary around the tank so that it can’t blow away anymore"* (Teacher, school 19), and *"[The SMC] also gave a speech about handwashing in the mothers assembly"* (Teacher, school 10). The situation at the three schools assigned a score of 0 reveal a different story where the SMC doesn’t participate in school sanitation in any way: *"the SMC is active only during meetings but not the rest of the time"* (field officer, school 12), and *"the SMC doesn’t do anything related to sanitation and handwashing"* (Teacher, school 15).

### Government support

We included both GPS and RNGPS schools in the analysis. GPS typically receive government funding for expenses such as teacher salaries and utility bills, while RNGPS usually need to cover these costs through other sources. In addition, there are two funds offered by the government: the contingency fund and the school-level improvement plan (SLIP) fund. All GPS, and some RNGPS, receive the contingency fund, which is usually 700 tk (app. 9 USD) per month and meant for photocopies and other managerial needs. The SLIP fund, intended for maintenance and school improvements, is typically 20,000 to 30,000 tk (app. 240–370 USD) for the year and is only provided to a portion of schools each year, including some RNGPS.

Four schools, coded as 1, were currently receiving the maximum government funding including both contingency and SLIP funding. School 10, an RNGPS, also has the SLIP fund, but was coded as 0.67 since they don’t have the contingency fund. Eight schools were coded as 0.33: three RNGPS and five GPS. These schools have access to contingency funding, which they use for minor maintenance needs out of necessity, despite the main purpose of the fund being for teaching related expenses. The remaining three schools, coded as 0, are RNGPS schools that receive no government funding.

### Maintenance plan

Criteria for having a maintenance plan emerged from the cases, specifically, the importance of having one dedicated teacher (or one for each gender) assigned to manage the toilets, and following a regular cleaning schedule. Accordingly, we coded schools with both of these characteristics as 1 and schools with a cleaning schedule but no singular, dedicated person responsible for carrying it out as a 0.67. This is based on theory and case knowledge that suggest that following a cleaning schedule is a positive condition, but may not be as effective if a specific teacher is not held accountable for executing it. There were no schools with a dedicated person responsible for toilet maintenance that did not have a cleaning schedule. Five schools were assigned a code of 1, such as school 4 where the head teacher describes clear responsibilities, saying that *"one teacher is responsible for the toilet monitoring and maintenance and another teacher is responsible for ring well monitoring and maintenance."* Students recognize these roles as well as girls from school 4 explain, *"there is an assigned teacher for the boys…and for the girls. The teachers always remind us about health, sanitation and hygiene issues.*" All five of these schools follow a cleaning schedule that is led or monitored by the dedicated teacher. An additional five schools, coded as 0.67, follow a regular cleaning schedule but there is not one specific teacher responsible for sanitation. There were six schools coded as 0 that do not have a specific teacher responsible for the toilets and, according to students, the cleaning schedule is rarely followed, if there is a schedule at all, as the boys at school 17 describe, *"We are bound to use the urinals because we have no option left. The toilets are only cleaned once or twice… only when visitors come to our school."* All case schools fell into one of these three categories and we did not utilize a score of 0.33 for this condition.

### Presence of a champion

We coded schools as 1 if teachers, students and/or the field officer identified someone as a champion and the research team felt they were the main source of sanitation activity at the school whose absence would likely lead to the discontinuation of these activities. In a few cases this was the dedicated teacher responsible for sanitation maintenance, though in most it was the head teacher or an SMC member. In the coding, an active team of teachers where no single person was identified as being the "cause" of the activeness was coded as 0.33. Examples of this scenario are schools 10, 16 and 19 where the teachers work as a team and coordinate well with the SMC, but if any one teacher left the school, activities would likely continue.

Following this coding scheme, schools 8 and 14 were coded as 1. The head teacher at school 14 was identified as a champion by students,*"Oh my gosh! If we forget to put soap [in the toilet] and madam finds out, she tells us to put it in. She asked us affectionately, 'Why didn’t you tell me? Did I ever say that I will not give you soap? Whenever you need soap just come to me’"* (focus group, boys), and the field officer, *"This school really works as a team with the lead of the head teacher."* The temporary teacher assigned by Save the Children at school 8 was identified by multiple students as a champion who said, "*We have a list of who should collect water when. [Teacher’s name] made the list"* (focus group, girls), and *"Yes, [Teacher’s name] talks to us about proper toilet use. … she taught us about handwashing"* (focus group, girls). Four schools were coded as 0.67 for having someone who takes action to improve sanitation at the school but who does not lead all the improvements needed. School 4 provides an example, where a teacher describes the SMC vice president as *"…very active in sanitation and hygiene issues… When [name] comes to see the school, first he checks the toilet. If it is dirty, he starts to clean it himself."* A score of 0.33 was assigned to five schools where there is someone interested in school sanitation, but they have taken only limited or infrequent action or their departure would likely have little effect on the continuation of activities, such as school 13 where *"…the head of the SMC is very active all year long. He visits the school every month and talks with the students about health and sanitation…"* (Teacher). At the remaining six schools, no champion was identified by teachers, students, the field officer, or data collectors: e.g. *"there is no teacher that is responsible for sanitation and hygiene at the school and no one from the village is very involved"* (Teacher, school 1).

### Pathways to well-managed school sanitation services

**Figure 1:**
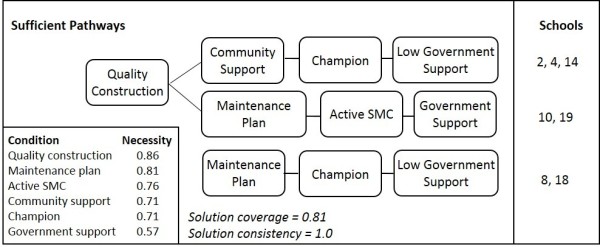
**Pathways to well-managed school sanitation services in Bangladesh.** Three pathways are shown where each series of lines between conditions indicates a combination of conditions that are significant to lead to well-managed school sanitation. Each pathway explains the case schools listed in the right column, but the number of case schools does not imply weighting, as each pathway is considered sufficient. The first two pathways explain both government and non-government schools, while the third explains non-government schools only. Results from necessity analysis of each individual condition are presented in the lower left box, where a score of 0.9 or higher is needed to signify necessity. The solution coverage indicates that 81% of set memberships in the positive outcome can be explained by these pathways, with a consistency of 1.0 meaning that 100% of the cases with the characteristics of at least one of these pathways have well-managed sanitation.

Analysis of the case schools with well-managed sanitation reveals three sufficient pathways, as shown in Figure 1, where the lines between conditions represent a pathway and each pathway is considered sufficient to produce the outcome [19,21]. One of the pathways has a combination of the following four conditions: quality construction, financial support from the community, the presence of a local sanitation champion, and the absence of SLIP funding from the government. Another pathway combines quality construction, the presence of a maintenance plan, an active SMC, and current government SLIP funding. And, finally, identifying the common conditions among the two schools with poor quality construction, the last identified pathway has a maintenance plan, a local sanitation champion, and low government support (contingency fund only). The pathways are presented in no particular order as each represents a sufficient combination of conditions.

Based on necessity scores, none of the individual conditions meet the cut-off of 0.90 to be considered "necessary". However, if we run necessity analysis on community support *or* government support (evaluating if *either* community or government support is present in 90% or more of the cases with the desired outcome), we find that financial access, from either of these two sources, is necessary with a score of 0.90. This is further reflected in an independent cost analysis of the *Shishuder Jonno* program which highlighted the need to transfer maintenance costs from Save the Children to the government and/or community [32]. Though important, financial access alone is not sufficient for well-managed services, as illustrated at school 1, where a teacher said *"we have a strong fund from the government and we don’t spend all the money in a year. So we always have money for maintaining,"* yet the toilets are frequently broken down and they were waiting for Save the Children to repair a broken pipe. Hence, other conditions are needed to create the motivation to utilize available funds to create reliably functioning and clean sanitation services to students, as seen in the pathways.

It is interesting to note the *absence* of government SLIP funding in the first and third pathway. We hypothesize two reasons for this from further analysis of the case schools. One, RNGPS, which normally receive little to no government support, tend to have very active and independent teachers as exemplified by a teacher at school 16, *"We are a non-government school. We built this school and we are running it. We paid for everything."* Teachers at RNGPS are often motivated to create a positive school environment so that parents continue to send their children and the school is eventually given GPS status. Two, government funding is described by teachers as delayed and distributed at random, restricting planning and quick recovery from breakdown at schools that depend primarily on government support, as teachers explain that *"The government takes a long time to process the funding. We don’t get the money in due time"* (school 3), and *"If we go to the government office, the process will be like: you apply for a blanket in the winter, they will give it to you in summer. It takes a season to repair with government involvement"* (school 16).

Both of the schools explained by the second pathway have a toilet cleaning schedule with one dedicated teacher responsible for sanitation (coded as 1) and the SMC is highly active, including rapidly responding to repair needs identified by the teachers and talking with students and parents about hygiene (coded as 1). As the only pathway without reliance on an individual sanitation champion, the second pathway may provide insight into a more robust option than pathways 1 and 3.

The nationalization of all schools in 2013 may have implications for the sufficiency of pathways that explain only RNGPS. Pathways 1 and 2 each explain both GPS and RNGPS case schools, implying that as RNGPS nationalize, these two pathways are likley to remain sufficient. However, the two schools explained by the third pathway are RNGPS and the sufficiency of this pathway may not hold post-nationalization. Beyond the longevity concerns as RNGPS convert to GPS, the generalizability of this pathway may be limited, as financial access is not present, particularly as more time passes and repair needs become more costly. Looking deeper at the case data, the moderate success of school 8, with an outcome score of 0.67, is likely dependent on the temporary teacher who is partially funded by Save the Children and has been very active in promoting sanitation and hygiene at the school, and the success of school 18 is likely due to financial support from the community, a very active SMC, and a champion head teacher.

Considering the potential tenuity of pathway 3, two options are presented (pathways 1 and 2) depending on the local context. If adequate financial support from the community for maintenance cannot be secured and there is no reliable champion, the conditions in pathway 2 may present more realistic areas to focus resources; and, vice-versa, if the school does not have government SLIP funding, then focusing on the conditions in pathway 1 may be more effective. Only some schools receive SLIP funding each year, suggesting that either government funding will need to increase to provide all schools with SLIP funding (in addition to encouraging SMC involvement and a dedicated teacher for school sanitation), or community support and a champion will be needed at the schools without current access to this fund.

### Pathways to poorly managed school sanitation services

**Figure 2:**
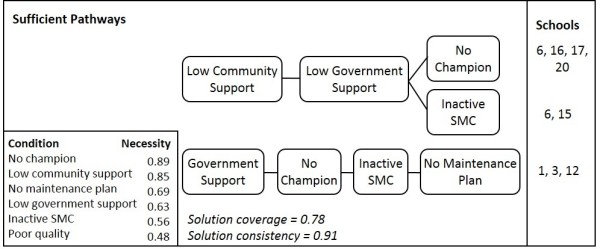
**Pathways to poorly managed school sanitation services in Bangladesh.** Three pathways are shown where each series of lines between conditions indicates a combination of conditions that are sufficient to lead to poorly managed school sanitation. Each pathway explains the case schools listed in the right column, but the number of case schools does not imply weighting, as each pathway is considered sufficient. The first two pathways explain both government and non-government schools, while the third explains government schools only. Results from necessity analysis of each individual condition are presented in the lower left box, where a score of 0.9 or higher is needed to signify necessity. The solution coverage indicates that 78% of set memberships in the negative outcome can be explained by these pathways, with a consistency of 0.91 meaning that 91% the cases with the characteristics of at least one of these pathways have poorly managed sanitation.

Analysis of case schools with poorly managed sanitation confirm findings from the analysis of schools with well-managed services. Three sufficient pathways to poorly managed services are identified (Figure 2). Two pathways demonstrate the negative effect of insufficient financial access (from the government or community), where the absence of a champion or an inactive SMC combined with limited financial support is sufficient for poorly managed sanitation. The other pathway suggests that schools with government funding that have an inactive SMC, no maintenance plan and no champion are unlikely to provide reliable sanitation services. All three of the case schools explained by this pathway are GPS and though they have substantial financial government support through SLIP funding, there may be little motivation for teachers to maintain sanitation services without pressure from a champion, an active SMC, or the motivation of RNGPS teachers to "prove" their ability to run a quality school.

### Identifying underlying barriers and opportunities

The in-depth data collected during this study facilitate the identification of underlying reasons for the presence or absence of influential conditions, providing insight into *how* to encourage the pathways to well-managed services identified in the results.

#### Quality construction

Though the majority of the toilets were high quality, Save the Children staff described challenges with some contractors who compromised quality to reduce costs. Based on teacher feedback, frequent monitoring by the Save the Children engineer encouraged high quality construction. However, program engineers are unable to monitor the entire process at every school and additional local monitoring was common in the schools with high quality construction: *"If something breaks, it is…not the fault of construction or materials, because we checked the materials"* (Teacher, school 12). Save the Children encouraged local monitoring and many teachers felt their concerns were respected and acknowledged, such as at school 7 where a teacher said, *"The contractor brought a van of poor quality bricks, but we complained to the engineer about it and he forced the contractor to return the bricks and use new bricks for the construction."* Despite this, there were some schools that were not open during construction, or where teachers and parents felt uncertain of how to monitor construction or placed limited importance on sanitation.

#### SMC involvement

According to teachers and field officers, barriers to SMC activity include that members are busy as explained by a teacher at school 3, *"The SMC doesn’t have time to visit the school,"* and personal conflicts between teachers and SMC leadership as described by the field officer for school 19, *"The president of the SMC resigned 9–10 months ago because of personal problems between him and the head teacher… Six months ago, the SMC was not active, but now it is better."* Other case schools provide examples of how it may be possible to "activate" the SMC. At school 16, the head teacher contributes the recent increased activity of the SMC to a training session conducted by Save the Children and the local government, which focused on sanitation and hygiene themes during the third and final day. Teachers at school 4 shared experiences with community members from their visit to a school awarded best school in the sub-district by the government and said, *"At the beginning, the villagers, even the SMC, didn’t show interest in the school. We showed the villagers the activity* (the school visit) *using a projector in the schoolyard. Only then the villagers and the SMC were very interested in the school."*

#### Government support

Regarding financial support, a number of schools said they did not know when they would receive SLIP funding next which made it difficult to plan for repair needs, With respect to government involvement, all case schools reported frequent visits from the assistant district or sub-district education officers, ranging from every two weeks to three months, to check attendance records and lessons. Unfortunately, sanitation and hygiene are meagerly included in inspections, if at all, as described by one teacher who says, *"[The assistant sub-district education officer] visits our school once a month, but they don’t check anything related to the toilets or handwashing."* Though toilet cleanliness is sometimes inspected, only school 4, who has well-managed sanitation, felt sanitation and hygiene were prioritized by the education officer, saying *"He gave priority to the health and sanitation issue…"* when describing education officer visits.

#### Community support

Teachers report that they often have trouble securing financial contributions from the community: *"The villagers don’t participate financially, that’s the greatest challenge for funding toilet maintenance"* (Teacher, school 8). Even schools where teachers feel parents would contribute, they express hesitation in asking: *"We feel embarrassed to ask the villagers for the money"* (Teacher, school 16). However, households’ willingness to pay for community water services suggests potential for school sanitation support. In many communities, families contribute 10 to 20 tk (app. 0.13-0.25 USD) per month to maintain community arsenic treatment units (R. Mallik, personal communication, January 10, 2013). Based on an average student population of 271 and assuming two school-aged children per family, this would amount to 203–406 USD per year, similar to the SLIP fund. As seen at school 4, community financial support may be influenced by the feeling of inclusion and social pressure associated with teachers sharing their experiences from a visit to the best school in the sub-district (described previously), as one teacher explains, *"After that, whenever we ask the students to bring extra money for school activities the parents are willing to pay it."* The SMC could also be a source of advocacy in the community and a number of teachers reported their positive influence, such as the teacher at school 14 who says, *"In the local community, we use the SMC to raise awareness about sanitation and handwashing. The SMC members also live here so they can influence the people.*"

#### Maintenance planning

It should be noted that the presence of a cleaning schedule does not guarantee the schedule will be followed, as expressed in the Little Doctor focus group at school 10, "*Our teacher made some groups for toilet cleaning, but the fact is, sometimes the other group…doesn’t clean the toilet,"* and monitoring by a teacher is likely necessary such as at school 15: "*We need to monitor though when [the students] clean the toilet."* Monitoring student cleaning and repair needs was much more common at schools where one teacher was responsible for sanitation, usually appointed by the field officer or head teacher.

#### Local sanitation champions

The champion teacher at school 15 was identified as *"…the only local teacher. The others are not from the community and don’t really care"* (field officer, school 15), suggesting that local teachers may be more likely to take on a champion role. School health competitions for SMC members may also cultivate champions as described by the teacher at school 4, "*There is an SMC member… He is very active in sanitation and hygiene. He placed first among the whole upazila [sub-district] and zila [district] for the activity."* On the other hand, teacher transfer may remove champions, as girl students from school 3 explain, *"When we were in grade 4 we had a teacher… but he transferred to another school. Since then no one talks to us about handwashing."* To an extent, the field officers are all acting as champions, and though active field officers can be a positive influence, caution may be needed to discourage schools from relying on them. Field officers that are seen as the school’s champion may actually hinder long-term sustainability if the focus on sanitation departs with them, either when they leave for the day, or at the end of the Save the Children program: *"if teachers believe in hygiene and act accordingly, it will work, but if they only do things when the field officer comes, it won’t"* (field officer, school 11). There is a tendency for some field officers to want to be seen as a champion, as one field officer explains, "*it’s really the field officers work and the field officer should have the credit."* Though normally very positive, this aspiration may hinder the continuation of activities if sanitation leadership is not transferred to the teachers and SMC, as expressed by another field officer, *"I will not be here long term, but if somehow I can manage to get the SMC involved with the program, it will run for a longer time."*

## Discussion

Based on 16 case schools in Meherpur, Bangladesh, with varying levels of sanitation service conditions, this research identified two distinct pathways sufficient to support well-managed services that are applicable to both government and non-government schools: (1) quality construction, financial community support and a champion; and (2) quality construction, financial government support, a maintenance plan and school management committee involvement. Based on these findings, on-going financial support for operations and maintenance is found to be a *necessary* condition for continued management of school sanitation. This effect was particularly strong with financial support from the community, potentially due to the strength of champions at non-government schools and the inconsistency of government funding. However, financial support was insufficient alone and other motivating conditions are needed, including quality construction (where poor quality can demotivate adequate management due to frequent maintenance needs) and incentivizing conditions, such as an SMC that is not only active, but involved in sanitation specifically, a local sanitation champion, and having one teacher that is held accountable for the school toilets, including a clear maintenance plan. Surprisingly, the number of students per toilet (ranging from 18–95 students) and toilet age (ranging from 8–32 months) had no significant effect on the condition of sanitation services.

Comparison of results from Bangladesh to those of a similar study in Belize [16] corroborates the need for community support and the tenuous reliance on champions in the absence of adequate government support. Results presented in this study, where government funding varied between schools, expand upon this finding to suggest that schools that do have government support still require a source of motivation to maintain services, such as SMC involvement in sanitation and a maintenance plan.

Further analysis revealed a number of potential sub-determinants of the conditions in the pathways to well-managed school sanitation, including that quality construction was more common among schools with local construction monitoring by teachers and/or parents, and schools where the Save the Children field officer was encouraging teachers and parents to play the role of champion, rather than acting as the champion themselves, helped to create and reinforce local sanitation champions. One school also noticed greater SMC involvement and financial support from the community after the teachers shared experiences from their visit to a school awarded "best school in the sub-district" by the government, suggesting that the social pressure created through this activity may have a positive influence on local involvement and support.

### Study limitations

Conditions that were constant among the case schools were excluded from analysis and should be considered when evaluating the generalizability of findings and in future studies. These include, but are not limited to, the national policy environment, local involvement in planning and construction, the technology installed (pour-flush toilets to septic tank), external monitoring by field officers multiple times per week, weekly hygiene classes including information on proper toilet use and handwashing with soap which all student focus groups could recall, and 98% of intervention cost funded by an NGO, with the government of Bangladesh covering the remaining 2% [32].

Additionally, three conditions were excluded due to limited variation: vandalism, water scarcity, and Little Doctor activity. However, the exclusion of these conditions does not appear to impact results. The schools with vandalism (schools 1 and 17), water scarcity issues (schools 3, 10, and 17), and less active Little Doctors (schools 1 and 17) had numerous other low scoring conditions and none of these schools can be explained by any of the three pathways to well-managed services identified. The other conditions present (or absent) are also in line with other schools with poorly-managed sanitation services and it is unlikely that removing the vandalism or water scarcity issues alone would result in well-managed sanitation services at these schools. However, these challenges deserve further attention as they have the potential to hinder improvement in other areas if not addressed.

A further limitation is that data were collected from one point in time and the condition of facilities on the day of the research visit may be atypical. However we attempted to capture any deviation through student focus group discussions and teacher interviews, which provided a longitudinal perspective through answering questions regarding past downtimes in service provision and average cleanliness.

## Conclusions

We identified two pathways (combinations of conditions) sufficient for well-managed school sanitation, that were absent in schools with poorly managed sanitation, and are applicable to both government and non-government schools. The pathways support the conclusion that a combination of on-going financial support for operations and maintenance and incentivizing conditions are needed to realize long-term management of school sanitation investments.

Potential sub-determinants of the conditions were then identified through further analysis of in-depth qualitative data.

These findings may have broader implications for school sanitation in other low-income countries, and institutionalizing structures that foster the conditions identified in the pathways to well-managed services could bring these lessons to scale both within and outside of Bangladesh. However, further investigation to verify and expand on results in another geographical and cultural context is needed. In particular, more research is needed regarding how to encourage the conditions observed in the pathways to well-managed services, specifically with respect to streamlined and reliable financial support for maintenance.

## Abbreviations

csQCA: Crisp-set qualitative comparative analysis; fsQCA: Fuzzy-set qualitative comparative analysis; GPS: Government primary school; NGO: Non-government Organization; QCA: Qualitative comparative analysis; RNGPS: Registered non-government primary school; SLIP: School Level Improvement Plan (government fund); SMC: School management committee; USD: United States Dollar.

## Competing interests

Save the Children funded travel costs for CC and LB, and travel costs and a small stipend for KA. However, the results were not tied to any potential gains or losses and the authors were encouraged to present complete findings and express honest opinions. MV is a full-time employee of the organization, but is based in India and did not directly implement the program in Bangladesh. Additionally, transparency is paramount in her role to provide technical oversight to improve program quality and support evaluations to assess program efficacy and sustainability in the region. To avoid any subconscious bias in the organization’s favor, she was not involved in the collection, management or coding of raw data. She reviewed the accuracy of findings regarding the intervention program, but authors from the University of Colorado were given the final decision on the information to publish, which was not tied to any potential financial or non-financial gains or losses.

## Authors’ contributions

CC designed and coordinated the study, analyzed the data and drafted the manuscript. AJW provided guidance for the data collection, analysis and interpretation of the data and contributed to the manuscript. KGL provided guidance on the overall study, reviewed the research protocol and contributed to the manuscript. KA conducted interviews and focus groups, reviewed coding definitions, and worked with CC to conduct inter-rater reliability tests. LB collected all observational data and home visits, supported KA during focus groups, and worked with CC to clean the data and conduct inter-rater reliability tests. MV supported the study design including development of data collection tools and the study protocol, and contributed to the final manuscript. All authors read and approved the final manuscript.

## Pre-publication history

The pre-publication history for this paper can be accessed here:

http://www.biomedcentral.com/1471-2458/14/6/prepub
